# Nuclear p120-catenin regulates the anoikis resistance of mouse lobular breast cancer cells through Kaiso-dependent Wnt11 expression

**DOI:** 10.1242/dmm.018648

**Published:** 2015-02-20

**Authors:** Robert A. H. van de Ven, Milou Tenhagen, Wouter Meuleman, Jeske J. G. van Riel, Ron C. J. Schackmann, Patrick W. B. Derksen

**Affiliations:** 1 Department of Pathology, UMC Utrecht, 3584 CX Utrecht, The Netherlands.; 2 Division of Gene Regulation, Netherlands Cancer Institute, 1066 CX Amsterdam, The Netherlands.; 3 Division of Molecular Biology, Netherlands Cancer Institute, 1066 CX Amsterdam, The Netherlands.; 4 Faculty of Electrical Engineering, Mathematics and Computer Science, Delft University of Technology, Mekelweg, 2628 CD Delft, The Netherlands.

**Keywords:** p120-catenin, Kaiso, Breast cancer metastasis, Anoikis resistance

## Abstract

E-cadherin inactivation underpins the progression of invasive lobular breast carcinoma (ILC). In ILC, p120-catenin (p120) translocates to the cytosol where it controls anchorage independence through the Rho-Rock signaling pathway, a key mechanism driving tumor growth and metastasis. We now demonstrate that anchorage-independent ILC cells show an increase in nuclear p120, which results in relief of transcriptional repression by Kaiso. To identify the Kaiso target genes that control anchorage independence we performed genome-wide mRNA profiling on anoikis-resistant mouse ILC cells, and identified 29 candidate target genes, including the established Kaiso target *Wnt11*. Our data indicate that anchorage-independent upregulation of *Wnt11* in ILC cells is controlled by nuclear p120 through inhibition of Kaiso-mediated transcriptional repression. Finally, we show that Wnt11 promotes activation of RhoA, which causes ILC anoikis resistance. Our findings thereby establish a mechanistic link between E-cadherin loss and subsequent control of Rho-driven anoikis resistance through p120- and Kaiso-dependent expression of *Wnt11*.

## INTRODUCTION

E-cadherin is the gatekeeper of epithelial integrity by linking its extracellular homotypic cis and trans interactions to the actin and microtubule cytoskeleton through β-catenin, α-catenin and p120-catenin (p120) in cellular structures called adherens junctions (AJs) (reviewed in Gumbiner, 2005). Loss of AJ function through inhibition of E-cadherin was linked to cancer cell invasiveness more than two decades ago (Vleminckx et al., 1991), and these findings were further substantiated shortly thereafter by the discovery of inactivating germ line and somatic E-cadherin mutations in gastric carcinoma and breast cancer (Berx et al., 1995; Guilford et al., 1998). In breast cancer, somatic inactivation of E-cadherin has been causally linked to the development and progression of invasive lobular carcinoma (ILC), a main breast cancer subtype comprising ~15% of all breast cancers (Berx et al., 1995; Derksen et al., 2006; Derksen et al., 2011). ILC is characterized by noncohesive and infiltrative growth patterns, which render clinical diagnosis difficult when using standard X-ray mammography (Arpino et al., 2004). Genome-wide gene expression profiling has indicated that ILC forms a distinct breast cancer subtype based on hierarchical clustering, which implies that tumor development occurs through distinct genetic pathways (Korkola et al., 2003). Furthermore, ILC is characterized by downregulation of genes involved in actin cytoskeleton remodeling, cell adhesion and TGF-β signaling, whereas genes implicated in transcriptional regulation of immediate early genes and cell migration are upregulated (Weigelt et al., 2010).

In breast cancer, loss of E-cadherin has differing consequences for AJ-associated catenins. For example, β-catenin is rapidly degraded by the proteasome upon loss of E-cadherin and does not hyperactivate the canonical Wnt signaling pathway (Herzig et al., 2007; Schackmann et al., 2011). Although in normal epithelium p120 controls stability of the AJ through binding and stabilization of E-cadherin (Davis et al., 2003; Ireton et al., 2002; Nanes et al., 2012), loss of E-cadherin leads to translocation of p120 to the cytosol where it acts as an oncogene by regulating anchorage-independent tumor growth and metastasis through GTPase-dependent induction of resistance to anoikis (apoptosis that is induced through loss of cell-matrix adhesion) (Dohn et al., 2009; Schackmann et al., 2011; Soto et al., 2008).

Like other members of the Armadillo (ARM) domain protein family, p120 can shuttle between the cytosol and the nucleus (van Hengel et al., 1999). Additional examples include adenomatous polyposis coli (APC) (Neufeld et al., 2000), β-catenin (Behrens et al., 1996), importin-α (Görlich et al., 1994) and ARVCF (Mariner et al., 2000). Nucleocytoplasmic shuttling of p120 seems to be dependent on two distinct nuclear localization signals (NLSs), a nuclear export signal (NES) and the ARM domains (Kelly et al., 2004b; Roczniak-Ferguson and Reynolds, 2003; van Hengel et al., 1999). Evidence for a functional role of p120 in the nucleus emerged after identification of the transcriptional repressor Kaiso (ZBTB33) as a direct p120-binding partner (Daniel and Reynolds, 1999). Kaiso is a member of the zinc finger and broad-complex, tramtrack and bric-à-brac or poxvirus and zinc finger (BTB/POZ-ZF) family of transcription factors (reviewed in Kelly and Daniel, 2006). Unlike its BTB/POZ-ZF family members, Kaiso is thought to interact with target gene promoters through sequence-specific Kaiso-binding sites (KBSs; consensus sequence TCCTGCNA) and/or methylated CpG dinucleotides (Daniel et al., 2002). Recent structural analyses of Kaiso in complex with DNA have revealed that recognition of specific bases in the major groove of the consensus KBS sequence and methylated CpG sites is accomplished through hydrogen bonds between residues in the first two zinc fingers, whereas high-affinity binding requires binding of the third zinc finger to the minor groove (Buck-Koehntop et al., 2012). Interestingly, binding of p120 occurs in a region adjacent to the zinc finger domains of Kaiso and effectively disrupts the DNA-binding ability of Kaiso (Daniel and Reynolds, 1999; Daniel et al., 2002). Binding to p120 relieves Kaiso-dependent transcriptional repression, suggesting that there is a crucial role for p120 in regulating gene expression (Daniel et al., 2002; Kim et al., 2004; Park et al., 2005). In the context of cancer biology, only a limited number of Kaiso target genes have been identified thus far, including the matrix metalloproteinase matrilysin (*MMP7*) (Daniel et al., 2002), metastasis-associated gene 2 (*MTA2*) (Yoon et al., 2003), cyclin D1 (*CCND1*) (Park et al., 2005) and Wnt11 (*WNT11*) (Kim et al., 2004). Wnt11 is a mediator of noncanonical Wnt signaling and is involved in developmental processes through control of Kaiso-dependent expression (Kim et al., 2004). Recently, Wnt11-induced Wnt signaling has been identified as a major paracrine factor driving breast cancer invasion (Luga et al., 2012). Interestingly, the *WNT11* locus maps to 11q13, a region frequently found amplified in human breast cancer (Schuuring et al., 1992).

**TRANSLATIONAL IMPACT**

**Clinical issue**

For cancer cells to metastasize, they need to overcome apoptosis that is induced upon loss of cell-cell or cell-matrix adhesion (resistance to anoikis). Invasive lobular cancer (ILC) is a major breast cancer type that is characterized by a non-cohesive and infiltrative growth pattern due to E-cadherin (a transmembrane protein that controls cell-cell adhesion) inactivation and subsequent constitutive activation of Rho-Rock signaling. Although most ILC cases are sensitive to initial hormone antagonist treatment, ILCs show overall poor responsiveness to conventional chemotherapy once the hormone antagonists are unsuccessful. Because ILC in general lacks expression of human epidermal growth factor receptor 2 (Her2; a marker present in some breast cancer subtypes for which specific treatments are available), there is currently no targeted intervention for metastatic lobular breast cancer.

**Results**

In this paper, the authors establish a functional link between loss of E-cadherin and subsequent activation of a distinct transcriptional program through nuclear influx of p120-catenin (p120) in ILC. Using mouse model systems of human metastatic ILC, evidence is provided that ILC cells are prone to relief of Kaiso-dependent transcriptional repression upon transfer to anchorage independence. The authors show that *Wnt11* (a non-canonical Wnt signal) is a direct and p120-dependent Kaiso target gene that regulates ILC anoikis resistance through autocrine activation of RhoA, a crucial regulator of invasion and metastasis.

**Implications and future directions**

ILC forms a distinct breast cancer subtype with a distinct tumor etiology based on early inactivation of cadherin-based cell adhesion. This study provides a molecular mechanism of how loss of E-cadherin leads to a p120-driven and ILC-specific gene expression program. The current study defines new candidate Kaiso target genes in anchorage-independent mouse ILC that could contribute to the development of a targeted intervention for ILC. The identification of a p120-Kaiso-Wnt11-dependent autocrine activation of RhoA that drives anchorage independence underpins the use of Rho-Rock targeting as a therapeutic strategy in the treatment of metastatic ILC.

Here, we show that translocation of p120 inhibits Kaiso-mediated transcriptional repression in ILC. Moreover, we were able to show that the nuclear pool of p120 is increased in anchorage-independent mouse ILC (mILC) cells, a mechanism which could induce Kaiso target gene expression specifically in anchorage-independent ILC. Using anoikis-resistant mILC cell lines we now identify (candidate) Kaiso target genes including *Wnt11* in anchorage-independent mILC cell lines. Moreover, we show that autocrine Wnt11 signaling promotes anoikis resistance through activation of RhoA, a cardinal event in the progression of metastatic lobular breast cancer.

## RESULTS

### p120 localizes to the cytosol and nucleus in human and mouse ILC

**Fig. 1.**
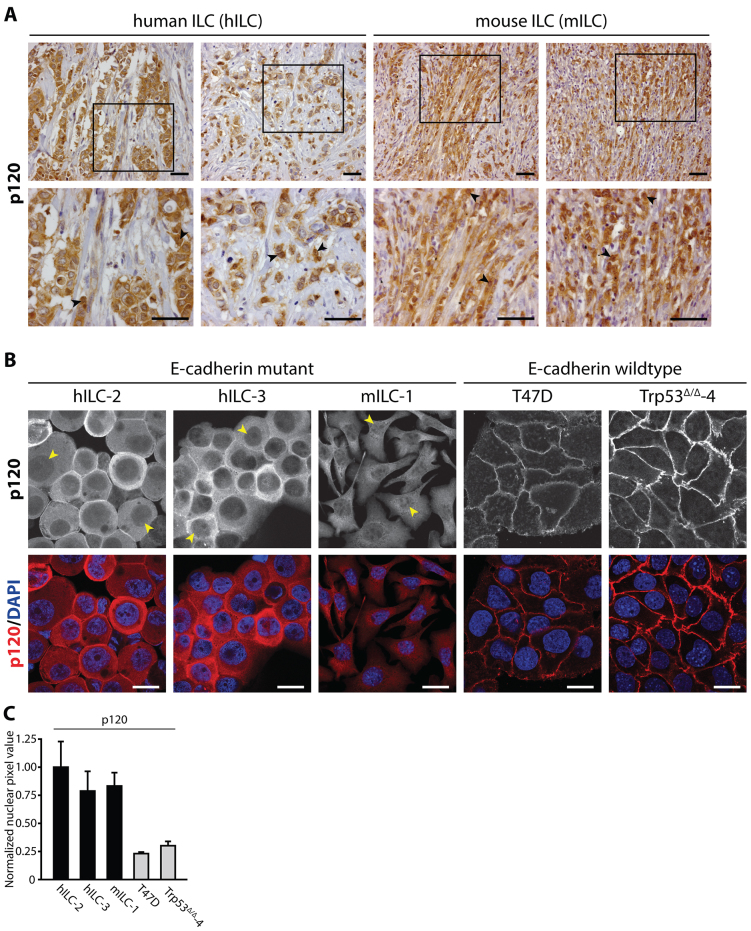
**p120 translocates to the cytosol and nucleus in mouse and human ILC cells.** (A) p120 localization in ILC. Immunohistochemistry showing expression of p120 in primary human ILC (left panels) and primary mouse ILC cells (right panels). Lower panels are magnifications that correspond to the area indicated in the upper panel. Arrowheads denote representative cells with pronounced nuclear p120 expression. Scale bars: 50 μm. (B) Nuclear p120 expression in primary metastatic human ILC and mouse ILC cell lines. Immunofluorescence staining for p120 (upper panels) shows nuclear and cytosolic p120 in the majority of two independent primary human metastatic ILC samples (hILC-2 and hILC-3) and the tumor-derived mouse ILC cell line (mILC-1). Arrowheads denote representative cells with nuclear p120 expression in E-cadherin mutant ILC. Lower panels show the merge with DAPI (blue). The human breast cancer cell line T47D and mouse mammary carcinoma cells (Trp53^Δ/Δ^-4) were used to exemplify the near absence of nuclear p120 in E-cadherin-expressing breast carcinoma cells. Scale bars: 10 μm. (C) Quantifications of nuclear p120 in cells shown in B. At least 10 cells per cell line were quantified. Results are expressed as mean±s.d.

Previously, we have generated mouse models for human metastatic ILC based on tissue-specific inactivation of E-cadherin and p53 using either cytokeratin 14 or whey acidic protein promoter elements to drive Cre recombinase (*K14cre;Cdh1^F/F^;Trp53^F/F^* and *WAPcre;Cdh1^F/F^;Trp53^F/F^*) (Derksen et al., 2006; Derksen et al., 2011). In these mouse models, loss of E-cadherin results in the translocation of p120 to the cytosol where it regulates anchorage-independent tumor growth and metastasis through p120-dependent activation of Rho-Rock signaling (Derksen et al., 2006; Schackmann et al., 2011). Because p120 has the ability to shuttle between the nucleus and the cytosol (Kelly et al., 2004b; Roczniak-Ferguson and Reynolds, 2003; van Hengel et al., 1999), we set out to determine a possible functional role for nuclear p120 in E-cadherin-negative breast cancer. We started by studying the extent of nuclear p120 influx in mouse and human ILC. Next to cytosolic p120 expression, we observed concomitant localization of nuclear p120 in human and mouse primary ILC tumors (Fig. 1A, arrowheads). Immunofluorescence analysis of cells derived from pleural effusion fluids confirmed that p120 was also localized to the cytosol and nucleus in primary human metastatic ILC (hILC-2 and hILC-3; Fig. 1B, arrowheads, a quantification of nuclear p120 is shown in Fig. 1C). In addition to cytosolic p120 localization, we also observed nuclear p120 in cells from our ILC mouse model (mILC-1; Fig. 1B,C, arrowheads). Human and mouse breast cancer cell lines T47D and Trp53^Δ/Δ^-4 are shown (Fig. 1B,C, right panels) to emphasize that cytoplasmic and nuclear p120 is virtually absent in nonmetastatic breast cancer cells expressing a functional E-cadherin-based adherens junction. From these data we conclude that, next to cytosolic translocation of p120, E-cadherin mutant ILC cells are characterized by nuclear p120 localization.

### Kaiso-dependent transcriptional repression is inhibited by nuclear p120 in mILC

**Fig. 2.**
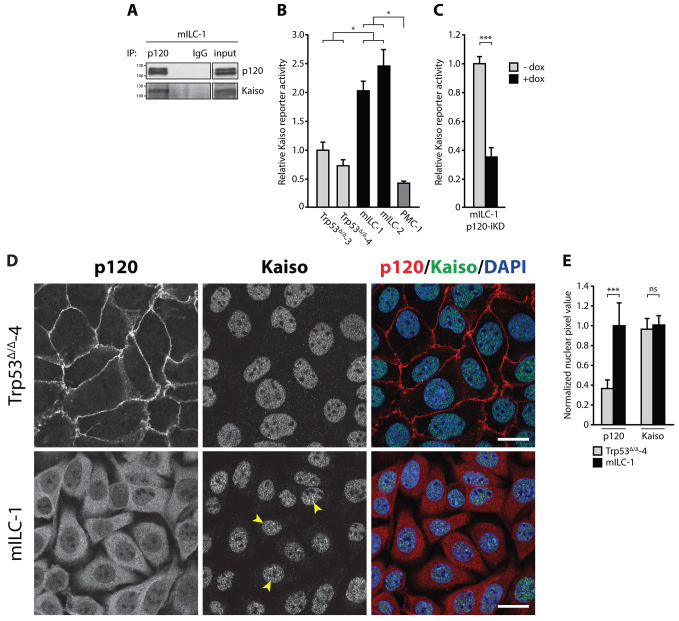
**Kaiso-mediated transcriptional repression is relieved by nuclear p120 in mILC.** (A) Kaiso interacts with p120 in mILC. p120 was immunoprecipitated to show the interaction between Kaiso and p120 (lane 1). Mouse IgG was used as a nonspecific immunoprecipitation (IP) control (lane 3). The total cell lysate (input) is shown in a separate panel. (B) Kaiso-mediated transcriptional repression is relieved in mILC. *Renilla*-based and Kaiso-specific reporter assays were performed on lysates from Trp53^Δ/Δ^, mILC and PMC-1 cells. (C) Nuclear p120 controls relief of Kaiso-mediated transcriptional repression in mILC. Findings in B were substantiated by inducible knockdown of p120 (p120-iKD) in mILC-1 cells. (D) Localization of p120 and Kaiso in the presence and absence of the epithelial adherens junction. E-cadherin-proficient Trp53^Δ/Δ^-4 cells and E-cadherin mutant mILC-1 cells were analyzed by immunofluorescence. Although p120 is predominantly localized to the plasma membrane in Trp53^Δ/Δ^-4 cells (upper panels), mILC-1 cells are characterized by cytosolic and nuclear p120 expression (lower panels). In contrast, the subcellular localization of Kaiso is not altered in mILC compared to Trp53^Δ/Δ^-4 cells. Note the punctate accumulation of Kaiso in mILC-1 nuclei (arrowheads). Scale bars: 10 μm. (E) Quantification of nuclear p120 and Kaiso in cells shown in D. Note the increase in nuclear p120 in mILC-1 compared to Trp53^Δ/Δ^-4. In contrast, no difference in nuclear Kaiso levels was observed between the two models. Shown are data from three independent experiments. Results are expressed as mean±s.d. *P<0.05; ***P<0.001; ns, not significant (Student’s *t*-test).

Previous studies have reported that nuclear p120 can interact and functionally antagonize the activity of the transcriptional repressor Kaiso. To corroborate these findings in breast cancer, we validated the interaction between p120 and Kaiso in mILC-1 cells by co-immunoprecipitation (Fig. 2A). Because p120 binds to Kaiso in a region near its zinc finger domains (an interaction that prevents Kaiso from functioning as transcriptional repressor), we hypothesized that nuclear influx of p120 could inhibit transcriptional repression by Kaiso and induce expression of Kaiso target genes in ILC. To functionally test this, we expressed a Kaiso reporter construct consisting of four repeats of the consensus KBS (4×KBS) (Kelly et al., 2004a) in a panel of mouse breast cancer cell lines. Indeed, Kaiso reporter activity was significantly higher in mILC cell lines compared to E-cadherin-expressing Trp53^Δ/Δ^ cell lines, indicative of relief of Kaiso-mediated transcriptional repression (Fig. 2B). To determine whether the observed elevated transcriptional activity was dependent on p120, we used a p120-deficient cell line (PMC-1), which we derived previously from a conditional mouse model of human metaplastic mammary carcinoma (*WAPcre;Ctnnd1^F/F^;Trp53^F/F^*) (Schackmann et al., 2013). Indeed, basal Kaiso reporter activity in PMC-1 cells was significantly reduced compared to mILC cell lines, suggesting that p120 antagonizes Kaiso-mediated transcriptional repression (Fig. 2B). To further substantiate these findings we transduced mILC-1 cells with a doxycycline (dox)-inducible p120-knockdown construct (p120-iKD), and observed a threefold decrease in Kaiso reporter activity upon dox administration (Fig. 2C). Taken together, these data demonstrate that translocation of p120 causes the inhibition of Kaiso-mediated transcriptional repression in E-cadherin-deficient ILC cells.

### Nucleocytoplasmic shuttling of p120 does not translocate Kaiso from the nucleus in ILC

Given the p120-dependent relief of Kaiso-mediated transcriptional repression in mILC cells, we reasoned that the nuclear influx of p120 could alter the subcellular localization of Kaiso. To test this, we initially assessed localization of p120 and Kaiso in mILC-1 cells and the E-cadherin-expressing Trp53^Δ/Δ^-4 cell line using immunofluorescence microscopy. Confirming previous results, we observed that Trp53^Δ/Δ^-4 cells showed p120 expression predominantly at the plasma membrane, whereas mILC-1 cells had both cytosolic and nuclear p120 localization (Fig. 2D, left panels). Interestingly, Kaiso localization was exclusively restricted to the nucleus independent of E-cadherin status, which suggests that the nuclear influx of p120 in mILC-1 cells does not alter the nuclear localization of Kaiso (Fig. 2D, middle panels). Quantification indeed confirmed these findings by showing that in contrast to p120, nuclear Kaiso levels were not affected by the presence or absence of E-cadherin (Fig. 2E). However, whereas total Kaiso levels were similar, we observed that in contrast to Trp53^Δ/Δ^-4 cells, mILC-1 cells displayed distinct nuclear foci of Kaiso (Fig. 2D, arrowheads).

**Fig. 3.**
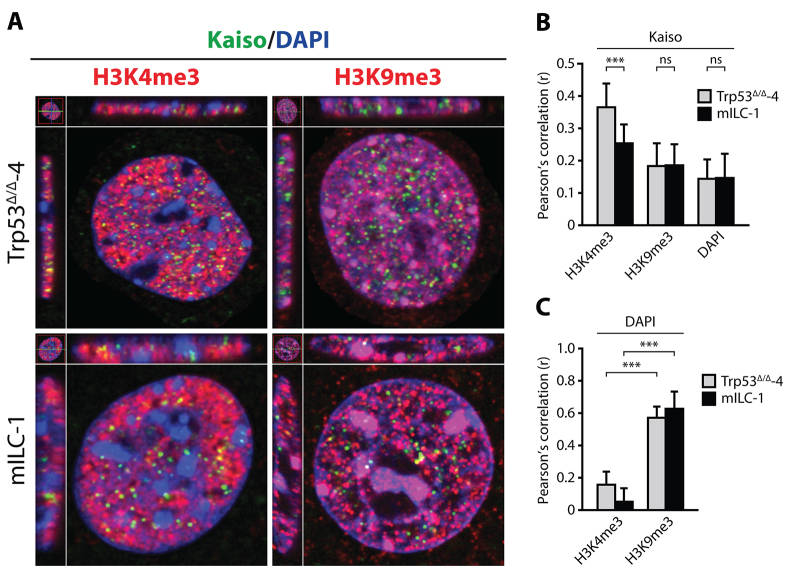
**Kaiso is displaced from genomic regions of active transcription in mILC cells.** (A) Colocalization of Kaiso with the active transcriptional mark H3K4me3, the inactive transcriptional mark H3K9me3 and heterochromatin (DAPI) was assessed by means of immunofluorescence. (B) Colocalization of Kaiso with the active transcription mark H3K4me3-positive genomic regions is decreased in mILC, whereas colocalization with the inactive transcription mark H3K9me3 is unaltered between cell models. Shown is a quantification of colocalization using the Pearson’s correlation coefficient (*r*). (C) Colocalization of DAPI with H3K4me3 and H3K9me3 was used as a validation of the method used in B. Results are expressed as mean±s.d. (*n*=40 or more analyzed nuclei). ***P<0.001; ns, not significant (Student’s *t*-test).

A recent report has suggested that there is a correlation between Kaiso binding to DNA and the presence of active transcription marks including H3K9ac, H3K27ac and H3K4me3 (Blattler et al., 2013). Therefore, we anticipated that nuclear p120 might alter Kaiso-mediated transcriptional repression through displacement of Kaiso from active promoter regions. By using immunofluorescence microscopy, we determined that colocalization of Kaiso and the active transcription marker H3K4me3 was significantly lower in mILC-1 cells when compared to Trp53^Δ/Δ^-4 cells (Fig. 3A–C). In contrast, colocalization of Kaiso and the inactive transcription marker H3K9me3 did not significantly differ between Trp53^Δ/Δ^-4 and mILC-1 cells (Fig. 3A–C). These findings suggest that Kaiso acts as a repressor on transcriptionally active genomic regions, a function that might be inhibited by the nuclear influx of p120. Our data also indicate that, although Kaiso is not exported from the nucleus, displacement of Kaiso from genomic regions associated with active transcription might underlie the observed relief of Kaiso-dependent transcriptional repression in ILC.

### Nuclear p120 is enriched in anchorage-independent mILC cells

**Fig. 4.**
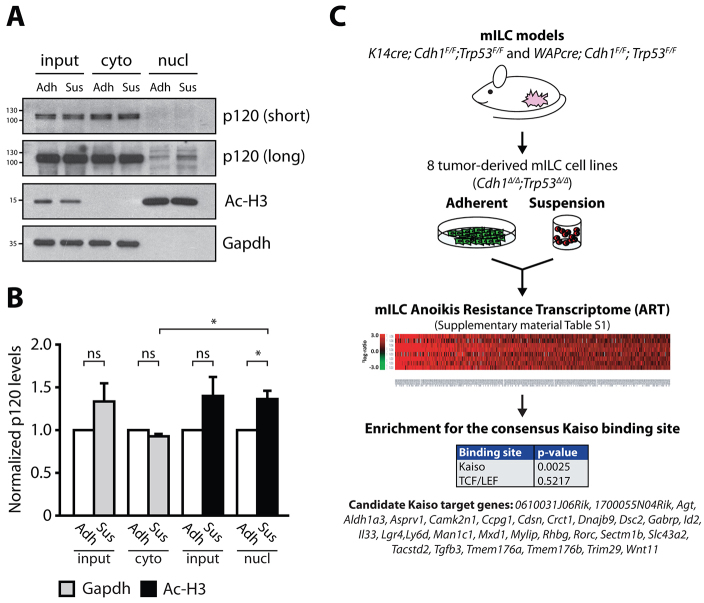
**Identification of candidate Kaiso target genes in anchorage-independent mILC.** (A) Nuclear p120 is enriched in anchorage-independent mILC cells. Fractionation was performed on adherent (Adh) and anchorage-independent (Sus) mILC-1 cells and the cytosolic and nuclear fractions were analyzed for p120 expression using western blotting. The purity of the lysate samples was confirmed by using the nuclear marker acetylated histone H3 (Ac-H3) and the cytosolic marker Gapdh (bottom panels). Subsequent analysis of the nuclear fractions showed enrichment for p120 in anchorage-independent mILC-1 cells (compare lane 5 and 6). Shown are two exposure times for the p120 blot (short and long). (B) Quantification of p120 in cytosolic and nuclear fractions. The signal for p120 was quantified and normalized to the appropriate marker (Gapdh for the cytosolic pool; Ac-H3 for the nuclear pool). Note the significant and specific increase in nuclear p120 in anchorage-independence compared to adherent conditions. Shown are the pooled data from three experiments. Results are expressed as mean±s.d. *P<0.05; ns, not significant (Student’s *t*-test). (C) The mILC Anoikis Resistance Transcriptome. To identify candidate Kaiso target genes specifically upregulated in anchorage-independent conditions, eight independent mILC cell lines were cultured in adherent and suspension and subjected to genome-wide microarray analysis. Using an arbitrary ^2^log1.5 cut-off we identified 249 genes that were upregulated in mILC cells cultured under anchorage-independent conditions. We termed this gene list the mILC Anoikis Resistance Transcriptome (ART). Subsequent TFBS promoter analysis of genes within the mILC ART revealed enrichment for the consensus binding sequence of Kaiso (KBS). No enrichment for TCF/LEF-binding sites was observed. Based on the presence of KBS sequences in the promoter regions of genes within the mILC ART we identified 29 candidate Kaiso target genes including *Wnt11*. The *P*-values reported in this figure are the result of initial testing for the KBS and TCF/LEF consensus sites by a Student’s *t*-test.

A hallmark of metastatic mILC cells is that they are able to overcome anoikis when grown in anchorage-independent conditions, a characteristic that can be used as a measure for *in vivo* metastatic potential (Derksen et al., 2006; Douma et al., 2004). Given the fact that membrane-uncoupled p120 controls anchorage-independent tumor growth and metastasis of ILC (Schackmann et al., 2011), and nuclear p120 antagonizes Kaiso-dependent transcriptional repression in mILC cell lines, we hypothesized that Kaiso target genes could contribute to anchorage-independent survival of metastatic ILC. In such a scenario, the rate-limiting step in the induction of Kaiso target gene expression would be the amount of nuclear p120 during anchorage independence. To test this, we performed cellular fractionations on cells cultured either in adherent conditions or in suspension, and were able to obtain pure cytosolic and nuclear fractions (Fig. 4A, lower two panels). Conforming to our hypothesis, we observed that anchorage independence induced a significant increase in nuclear p120 in mILC-1 cells (Fig. 4A, compare lanes 5 and 6; see Fig. 4B for quantifications). Although we did observe a non-significant increase in total p120 levels upon transfer to anchorage-independence, we did not detect an increase in the cytosolic p120 pool (Fig. 4B) which conforms to the notion that the increase in nuclear p120 is specific and is not merely the result of an overall upregulation of p120 in anchorage-independent mILC-1 cells. These results show that nucleocytoplasmic shuttling of p120 is increased in anchorage-independent ILC cells, and thereby provide a rational for the identification of Kaiso target genes in ILC specifically under anchorage-independent conditions.

### Identification of Kaiso target genes in anoikis-resistant ILC

To identify novel candidate Kaiso target genes specifically in anchorage-independent conditions, we harvested mRNA from eight independent mILC cell lines grown in the presence or absence of anchorage, and performed a genome-wide microarray analysis. These data were combined to generate a list of 249 genes that were upregulated in anchorage-independent mILC cell lines, based on an arbitrary ^2^log-ratio cut-off of 1.5. We termed this gene subset the mILC Anoikis Resistance Transcriptome (ART) (Fig. 4C; supplementary material Table S1). Subsequent gene ontology analysis revealed enrichment for genes involved in the regulation of apoptosis, cellular adhesion, proliferation and the immune response (supplementary material Table S2). Next, we performed transcription-factor-binding site (TFBS) analyses, which yielded a number of significantly enriched transcription-factor-binding sites (supplementary material Table S3). Interestingly, we identified enrichment of the consensus KBS (TCCTGCNA; *P*=0.0025) in the promoter regions of 29 genes (Fig. 4C), which is in agreement with our hypothesis that Kaiso activity is relieved in ILC. We did not detect enrichment of the TCF/LEF consensus sequences (*P*=0.5217), which is supported by work from our and other laboratories showing that loss of E-cadherin does not induce canonical Wnt signaling (Herzig et al., 2007; Schackmann et al., 2011). Importantly, we identified *Wnt11* among the list of candidate Kaiso target genes (Fig. 4C). *Wnt11* is a bona fide Kaiso target gene that has been linked to regulation of *Xenopus* gastrulation by Kaiso-dependent transcriptional repression (Kim et al., 2004).

In summary, we analyzed the transcriptional effects of anchorage independence in metastatic breast cancer cells and identified candidate Kaiso target genes. Our results suggest a functional link between anoikis resistance and p120-dependent relief of Kaiso-mediated transcriptional repression.

### Anchorage-independent upregulation of the Kaiso target gene Wnt11 is p120-dependent

**Fig. 5.**
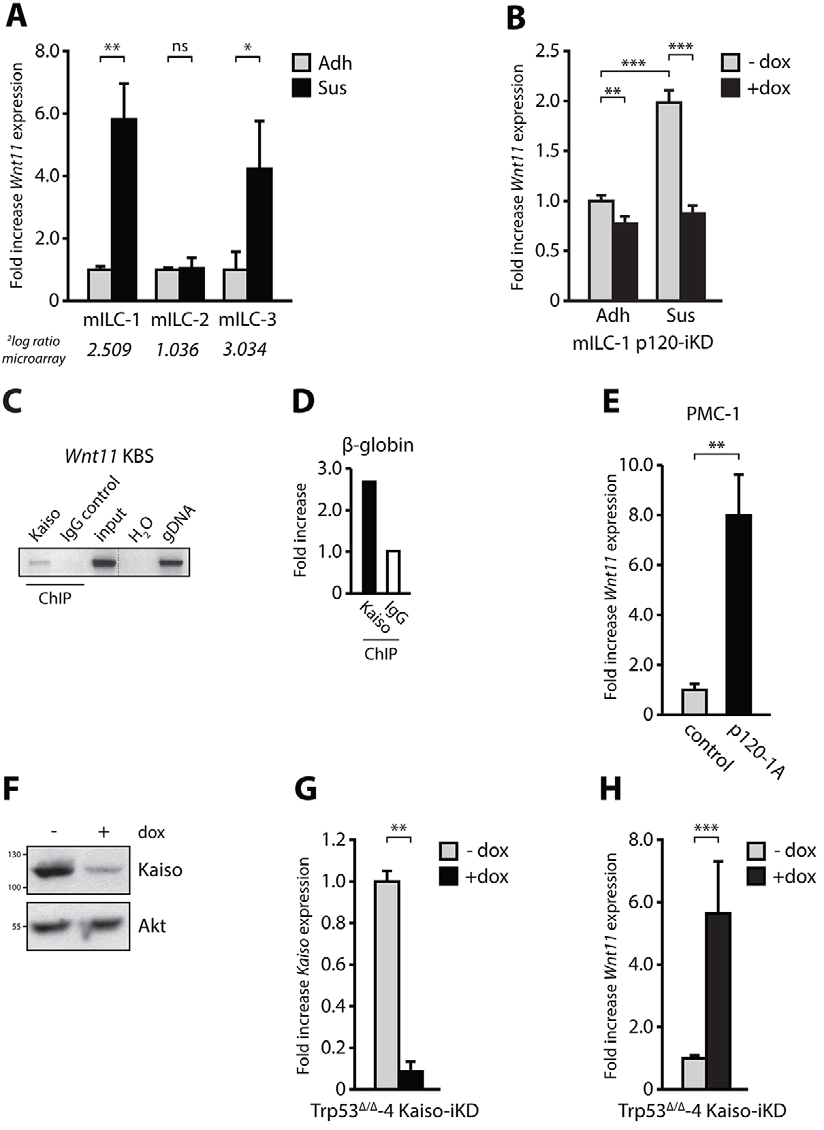
**Anchorage-independent upregulation of the Kaiso target gene *Wnt11* in mILC is p120-dependent.** (A) Anchorage-independent upregulation of *Wnt11* in mILC cell lines. Three independent mILC cell lines were cultured in the presence of cell-matrix anchorage (Adh) or in suspension (Sus). *Wnt11* expression was quantified using qPCR. No *Wnt11* upregulation was observed in mILC-2 cells, most likely due to high basal levels in anchorage-dependent conditions. The fold increase *Wnt11* expression derived from the microarray analyses (Fig. 3B) is shown in italic. (B) Upregulation of *Wnt11* is dependent on p120. *Wnt11* expression was assayed in mILC p120-iKD cells in the absence or presence of doxycycline (dox) and under adherent (Adh) and suspension (Sus) conditions using qPCR. (C,D) Kaiso binds to the *Wnt11* promotor. Kaiso-specific ChIP was performed on Trp53^Δ/Δ^-4 cells and mouse IgG was used as negative control (C). No input (H_2_O) and genomic DNA (gDNA) served as PCR controls. Specificity of the ChIP data shown in C was validated by amplification of a β-globin intronic promoter region (D). Fold increase of the KBS-specific signal compared to the β-globin signal was determined for the Kaiso-specific and the control ChIP using qPCR. (E) Expression of p120 in PMC-1 induces *Wnt11* expression. PMC-1 (Ctnd1^Δ/Δ^;Trp53^Δ/Δ^) cells were transduced with either a control vector or a vector expressing p120-1A. (F,G) Kaiso knockdown in Trp53^Δ/Δ^-4 Kaiso-iKD cells using western blotting (F) and qPCR (G). (H) Kaiso knockdown in Trp53^Δ/Δ^-4 Kaiso-iKD cells induces *Wnt11* expression. Shown are qPCR data from three independent experiments. Results are expressed as mean±s.d. *P<0.05; **P<0.01; ***P<0.001; ns, not significant (Student’s *t*-test).

Wnt11 is a key inducer of non-canonical Wnt signaling during vertebrate development and its expression is dependent on p120 and Kaiso (Heisenberg et al., 2000; Kim et al., 2004). In order to validate the anchorage-independent upregulation of *Wnt11*, we performed quantitative RT-PCR, and confirmed a transcriptional upregulation of *Wnt11* upon transfer to anchorage-independence in two out of three mILC cell lines (Fig. 5A). To assure that the anchorage-independent transcriptional upregulation of *Wnt11* is dependent on p120, we assayed *Wnt11* expression in adherent and anchorage-independent mILC-1 p120-iKD cells (Fig. 5B). Knockdown of p120 in adherent mILC-1 p120-iKD led to a twofold decrease in *Wnt11* levels in analogy with the decrease of Kaiso reporter activity shown in Fig. 2C. However, p120-iKD in the absence of anchorage reduced *Wnt11* expression to a level comparable to that in adherent mILC-1 cells (Fig. 5B), indicating a causal role for p120 in the control and increase of *Wnt11* expression in anchorage-independent ILC.

To confirm that p120 is indeed regulating *Wnt11* expression through Kaiso, we again made use of the E-cadherin-proficient Trp53^Δ/Δ^-4 cell line. We used this particular cell line because it showed low Kaiso reporter activity (Fig. 2B) and might therefore harbor the largest fraction of Kaiso bound to target gene promoters. Indeed, we were able to confirm binding of Kaiso to the *Wnt11* promotor in this cell line using chromatin immunoprecipitation (ChIP) and a PCR specific for the most proximal KBS in the mouse *Wnt11* promoter (Fig. 5C,D). To substantiate these findings, we probed the transcriptional consequences of introduction of p120 in the p120-deficient PMC-1 cell line. Conforming to our previous findings, expression of p120 indeed induced a dramatic increase in *Wnt11* levels (Fig. 5E). To confirm the causal role for Kaiso in repression of *Wnt11* expression, we assessed *Wnt11* expression in Trp53^Δ/Δ^-4 cells containing an inducible Kaiso short hairpin RNA (shRNA; Trp53^Δ/Δ^-4 Kaiso-iKD) and confirmed knockdown of Kaiso protein and mRNA (Fig. 5F,G). Ablation of Kaiso by administration of dox induced a threefold induction of *Wnt11* expression (Fig. 5H). In summary, these results confirm the validity of *Wnt11* as a genuine Kaiso target in mILC and demonstrate its regulation in E-cadherin-deficient breast cancer cells by nuclear p120.

### Wnt11 promotes RhoA-dependent anoikis resistance

**Fig. 6.**
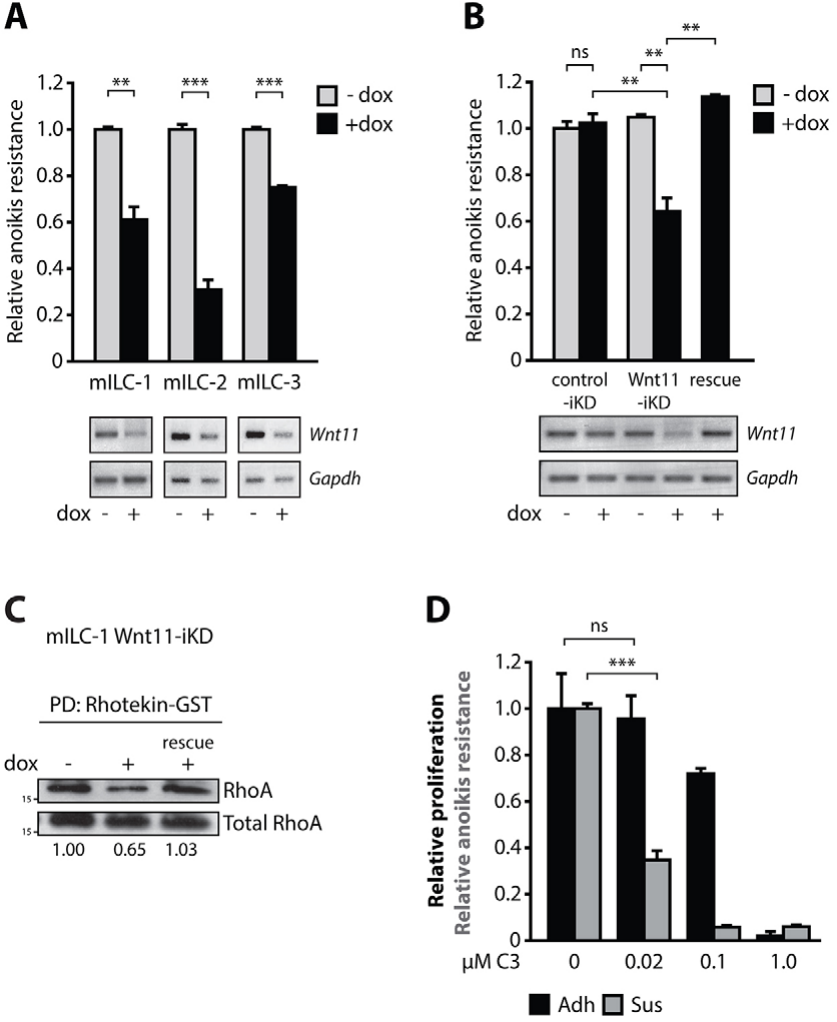
**Wnt11 regulates RhoA-dependent anoikis resistance in mILC.** (A) Knockdown of Wnt11 in mILC-1 Wnt11-iKD cells reduces anoikis resistance of anchorage-independent mILC cell lines. Knockdown of *Wnt11* was validated by RT-PCR. *Gapdh* was used as a loading control. (B) Introduction of a non-targetable Wnt11 cDNA in mILC-1 Wnt11-iKD restored anoikis resistance in mILC-1 cells (rescue). A scrambled shRNA (control-iKD; left lanes) was used as control for dox and shRNA expression. (C) Wnt11 regulates RhoA activation in mILC. Shown is a western blot depicting the effect of Wnt11-iKD on endogenous RhoA-GTP levels. GTP-loaded Rho was pulled down from the lysates using agarose-coupled Rhotekin-GST. Total RhoA was used as loading control. Note the rescue of Wnt11-iKD-induced RhoA inhibition (rescue). Shown below the blot are quantifications of RhoA-GTP levels. (D) Rho controls anoikis resistance of mILC cells. Inhibition of Rho by cell-permeable C3 transferase leads to inhibition of anoikis resistance of mILC-1 (Sus), but does not affect adherent cellular viability (Adh). Note the differential inhibitory effect at low molar concentrations. Shown are representative data from three independent experiments. Results are expressed as mean±s.d. **P<0.01; ***P<0.001; ns, not significant (Student’s *t*-test).

To investigate whether the p120-dependent upregulation of Wnt11 in mILC promotes anoikis resistance, we transduced three mILC cell lines with a Wnt11-iKD construct and assayed the effect on anchorage-independent survival in absence or presence of dox. Upon addition of dox, we observed a significant decrease in anoikis resistance in all mILC Wnt11-iKD cell lines (Fig. 6A). To demonstrate specificity of the used hairpin, we expressed a non-targetable Wnt11 cDNA in mILC-1 cells and observed a full restoration of the anoikis-resistant phenotype (Fig. 6B; right-most bar and lane). Wnt11 is known to mediate convergent extension movements during vertebrate gastrulation through activation of the small GTPase RhoA and its key downstream targets Rho-associated kinase (Rock) 1 and Rock2 (Marlow et al., 2002; Zhu et al., 2006). We recently showed that cytosolic p120 is a key regulator of ILC anoikis resistance through control of Rho-Rock activity by binding and inhibition of the Rho antagonist Mrip (Schackmann et al., 2011). Although the inhibition of Mrip by p120 provided a rationale for increased Rho activity in the presence of cytosolic p120, it did not reveal the upstream signal that drives RhoA. We therefore envisaged that Wnt11 could represent an autocrine cue that induces RhoA activation in mILC. To address this, we assayed RhoA activity in the absence or presence of Wnt11-iKD and observed that Wnt11-iKD resulted in a marked reduction of active GTP-bound RhoA (Fig. 6C, compare left and middle lanes), whereas expression of the non-targetable Wnt11 cDNA fully restored RhoA activity in these cells (Fig. 6C, right lane). In agreement with previous findings, we confirmed that RhoA activation was necessary for the anchorage- independent growth and survival of ILC cells (Schackmann et al., 2011). Treatment with the specific Rho inhibitor C3 transferase at a low molar concentration (0.02 μM) significantly reduced anoikis resistance in a dose-dependent manner, whereas adherent proliferation remained unaltered (Fig. 6D). In conclusion, our data indicate a crucial regulatory role for p120-Kaiso-dependent transcriptional regulation of Wnt11 and subsequent autocrine activation of Rho-dependent anoikis resistance in ILC.

## DISCUSSION

In order for cancer cells to metastasize, they have to overcome execution of apoptosis in the absence of cell-cell and/or cell-matrix interactions. This anoikis resistance is instigated upon mutational inactivation of E-cadherin, an initiating event that underlies the etiology of ILC (Berx et al., 1995; Derksen et al., 2006). Although our previous data identified p120 as an upstream driver of Rho-Rock-dependent anoikis resistance of ILC (Schackmann et al., 2011), the proximal cues controlling Rho activity remained unclear. Here, we show that nuclear p120 exerts an oncogenic function by inhibiting Kaiso-mediated transcriptional repression and thereby induces expression of the Kaiso target gene *Wnt11*, a factor that drives Rho-dependent anoikis resistance in ILC.

**Fig. 7.**
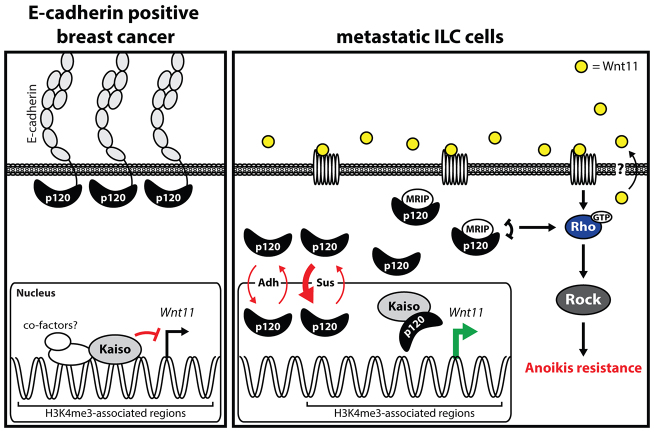
**A model for p120-dependent relief of Kaiso transcriptional Wnt11 repression in ILC.** In E-cadherin-expressing cancer cells, p120 resides at the plasma membrane in the adherens junction (AJ) complex with E-cadherin (left panel). In this scenario, Kaiso is repressing or dampening expression of its target genes in transcriptionally active regions by KBS-dependent binding and recruitment of repressive machinery. Upon loss of E-cadherin, p120 translocates to the cytosol and the nucleus (Adh), a shuttling process that is exacerbated by unknown mechanisms when cells lose cell-matrix interactions (Sus). In the nucleus p120 binds Kaiso and relieves Kaiso-mediated transcriptional repression. As a consequence, Kaiso target genes including *Wnt11* are expressed. Autocrine Wnt11 will subsequently activate the small GTPase RhoA, which is a crucial event in the regulation of anchorage-independent tumor growth and metastasis of ILC.

The presence of ARM domains in combination with nuclear import and export sequences renders p120 capable of shuttling in and out of the nucleus (Kelly et al., 2004b; Roczniak-Ferguson and Reynolds, 2003). Although it is still largely unknown how p120 transverses the nuclear pore and what factors mediate this transport, our observations support previous data showing that p120 relieves Kaiso-mediated transcriptional repression (Daniel et al., 2002; Kim et al., 2004; Park et al., 2005). Furthermore, we demonstrate that E-cadherin mutant breast cancer cells increase their nuclear p120 levels upon transfer to anchorage-independent conditions. Finally, our studies show that Kaiso is displaced from transcriptionally active genomic regions leading to increased endogenous Kaiso reporter activity in E-cadherin mutant breast cancer cells. Based on these findings we propose a model in which Kaiso functions as a general ‘brake’ that prevents or dampens transcriptional activity of genes in transcriptionally active genomic regions (Fig. 7). This mechanism would thereby function to induce rapid target gene expression upon (extra)cellular signals. As such, nuclear p120 acts as a rate-limiting antagonist of Kaiso-dependent transcriptional repression. Recently, a similar mechanism was proposed in the p120-dependent regulation of REST-CoREST-mediated transcriptional repression of genes involved in stem cell differentiation (Lee et al., 2014). Although the mode of action shared similarities, Kaiso did not associate with the p120-REST-CoREST complex, suggesting that nuclear p120 might have a broad function in the regulation of transcriptional repression.

ILC is renowned for diffuse infiltration patterns without overt desmoplastic responses. Interestingly, literature has reported several ILC cases in which distant metastasis had developed without the presence of a detectable primary tumor mass (Engelstaedter and Mylonas, 2011; Tomizawa et al., 2012), a phenotype we have also observed in the *WAPcre*-driven mouse models of human ILC (Derksen et al., 2011). Moreover, women suffering from ILC show a higher incidence in the development of contralateral lesions (Newstead et al., 1992) and long-term survival in ILC seems to be worse than other breast cancers when corrected for age and clinicopathological parameters (Korhonen et al., 2013). Given these characteristics, we hypothesized that disseminating ILC cells might have developed autocrine biochemical cues that promote cellular survival *ex situ*. By probing the transcriptional consequences of anchorage independence of eight independent mILC cell lines, we identified candidate Kaiso target genes as judged by the presence of the consensus KBS site in their promoter regions. These data are in line with observations described here and by others that cytosolic and nuclear translocation of p120 can relieve Kaiso-mediated transcriptional repression. In addition, we did not observe enrichment of genes harboring TCF/LEF-binding sites in their promoter regions confirming previous findings that the β-catenin-dependent canonical Wnt signaling pathway is not activated by loss of E-cadherin (Herzig et al., 2007; Schackmann et al., 2011). These findings not only demonstrate validity of our observations, they also suggest that, in an E-cadherin-deficient setting, biological responses through p120–Kaiso- and β-catenin–TCF-dependent transcriptional activity might be mutually exclusive. Given that Kaiso and TCF family transcription factors show overlap in their target repertoire (Park et al., 2005), we presume that specificity might have evolved in order to control homeostasis through differential regulation of a given gene in response to distinct proximal signals.

We identified *Wnt11* among the candidate Kaiso target genes and validated *Wnt11* as a direct Kaiso target gene in mILC. Research conducted in other laboratories has shown that Wnt11 plays important roles during vertebrate development through activation of the Rho-Rock signaling axis and subsequent cytoskeletal rearrangements (Heisenberg et al., 2000; Marlow et al., 2002; Zhu et al., 2006). Our data provide a novel concept in which loss of cadherin-based epithelial cell-cell adhesion drives activation of a specific promalignant transcriptional program. Moreover, we demonstrate that p120-dependent derepression of the Kaiso target gene *Wnt11* activates an autocrine Wnt11 signal that acts upstream of RhoA-dependent ILC anoikis resistance. Interestingly, several lines of research have indicated that Wnt11-mediated signaling acts as a player in breast cancer progression. For instance, stromal-derived paracrine Wnt11 signaling has been shown to transform mammary epithelial cells (Christiansen et al., 1996). In addition, estrogen related receptor α (ERRα)- and β-catenin-dependent Wnt11 expression has been shown to act as an autocrine promigratory cue in breast cancer cell lines (Dwyer et al., 2010). More recently, it has been demonstrated that Wnt11-loaded exosomes excreted by tumor-associated fibroblasts can activate the planar cell polarity signaling pathway and, through this mechanism, induce breast cancer metastasis (Luga et al., 2012). In short, although the mechanism underlying Wnt11 expression differs between studies, it is clear that induction of Wnt11 expression results in (pro)metastatic behavior of breast cancer cells. Taken together, we think our data have unraveled a mechanism whereby mutational inactivation of E-cadherin, and subsequent nuclear translocation of p120, leads to relief of Kaiso-mediated transcriptional repression of Wnt11 and subsequent anoikis resistance of ILC cells.

Although Wnt11-iKD induced a robust inhibition of mILC survival in suspension, a number of cells survived this condition, which could be explained by the observed Rho activity upon Wnt11-iKD. Our data do not exclude the possibility that residual Rho activity and subsequent survival might be caused by exogenous (serum-derived) Wnt11. Moreover, Wnt proteins are generally heavily glycosylated and can be retained at the plasma membrane by heparan sulfate proteoglycans (Tang et al., 2012). Given that this can result in highly localized ligand concentrations, small quantities will be sufficient to trigger the observed Rho activation upon Wnt11-iKD. Alternatively, residual Rho activity after Wnt11 inhibition can also be caused by intracellular cytoskeletal tension due to cellular rounding in suspension, which could potentially induce a positive feedback loop resulting in high Rho-GTP, as has been shown by others (Bhadriraju et al., 2007). Our previous and current research provides a clear rationale for the activation of Rock in ILC. First, somatic E-cadherin inactivation induces activation of Rock signals through p120-dependent binding and inhibition of the Rho antagonist MRIP (Schackmann et al., 2011). Second, current data show that activation of RhoA and Rock is maintained through a proximal autocrine activation of RhoA by Wnt11. Recent data have indicated that Wnt signals can be successfully inhibited by Frizzled receptor decoys and antibodies (Gurney et al., 2012), or prevention of Wnt secretion by the membrane-bound O-acyltransferase Porcupine (Liu et al., 2013). However, we believe that options for clinical interventions should be based on inhibition of Rho and Rock, because they represent the central hub in the regulation of anchorage-independent tumor growth and metastasis of E-cadherin mutant breast cancer.

In conclusion, our data establish a novel functional link between mutational inactivation of E-cadherin and subsequent p120-dependent relief of Kaiso-mediated transcriptional repression of *Wnt11*. In addition, we show that nucleocytoplasmic shuttling of p120 is the rate-limiting step in inhibition of Kaiso-mediated transcriptional repression. Subsequent anchorage-independent expression of the Kaiso target gene *Wnt11* drives anoikis resistance of metastatic breast cancer cells through activation of the Rho-Rock signaling pathway. This study thereby strongly advocates the potential of specifically targeting Rho-dependent Rock signaling as an intervention strategy in the clinical management of metastatic ILC.

## MATERIALS AND METHODS

### Cell culture and inhibitors

The origin of mouse models and culture of all tumor-derived mouse cell lines has been described previously (Derksen et al., 2006; Derksen et al., 2011; Schackmann et al., 2011; Schackmann et al., 2013). Transduced cells were cultured for 4 days in the presence of 1.0 μg/ml doxycycline in order to induce p120-, Kaiso- and Wnt11-specific shRNA expression (iKD). Cell permeable C3-transferase (Cytoskeleton, Denver, CO) was used according to the manufacturer’s instructions.

### Constructs, viral production and transduction

For stable knockdown of Wnt11 and Kaiso, shRNA sequences flanked by a *BbsI* and *XhoI* overhang (Wnt11 5′-TCCCGTCTGCAAGTGAGACC -ATATTCAAGAGATATGGTCTCACTTGCAGACTTTTTC-3′; Kaiso 5′-TCCCTCAGAAGATCATTACATTAGATTTCAAGAGAATCTAATGTAA -TGATCTTCTGATTTTTC-3′) were annealed and ligated into pH1tetflex (Herold et al., 2008). Subsequent cloning was performed as described previously (Schackmann et al., 2011). Creation of the p120-iKD construct was done as described previously (Schackmann et al., 2013). To generate the Wnt11 non-targetable construct, three silent point mutations were introduced in the pCR4-TOPO vector containing mouse Wnt11 cDNA (Thermo Scientific, Lafayette, CO) using site-directed mutagenesis (forward, 5′-GGAGCGCTACGTGTGTAAATGAGACCATATG-3′; reverse, 5′-CATA -TGGTCTCATTTACACACGTAGCGCTCC-3′). The resulting cDNA was cloned in the pLV.CMV.IRES.puro lentiviral vector using *Pst*I. Lentiviral production and transduction was done as described previously (Schackmann et al., 2011).

### Immunofluorescence microscopy

For immunofluorescence, cells were washed with PBS containing Ca^2+^ and Mg^2+^ and fixed with 4% paraformaldehyde for 30 minutes at room temperature. After washing, cells were permeabilized with 0.1% Triton X-100 in PBS for 10 minutes and blocked with 4% BSA in PBS for 10 minutes. Fixed samples were incubated with primary antibody for 1 hour at room temperature, washed with PBS and incubated with secondary antibodies for 1 hour at room temperature. Samples were stained with DAPI for 5 minutes and analyzed using a Zeiss LSM 700 (Carl Zeiss, Sliedrecht, The Netherlands). Images were processed using ImageJ and Adobe Photoshop CS6. For colocalization studies, image processing and quantification was done using Volocity (Perkin-Elmer, Waltham, MA). The following primary antibodies were used: mouse anti-Kaiso clone 6F (1:1000; a kind gift of Dr Juliet Daniel, McMasters University, Hamilton, Canada), mouse anti-p120-TRITC (1:200; BD Biosciences; #610137), rabbit anti-H3K9Me3 (1:500; Abcam; ab8898) and rabbit anti-H3K4Me3 (1:500; Diagenode; pab003-050) antibodies.

### Kaiso reporter assays

To generate pGL3-4XKBS-RLuc, *Renilla* luciferase (RLuc) was isolated from pRL (Promega, Madison, WI) by means of PCR (forward, 5′-AGCT -CCATGGCTTCGAAAGTTTATGATCCAGAACAAAGG-3′; reverse, 5′-TGGTCTAGAATTATTGTTCATTTTTGAGAACTCGC-3′) and ligated into pJET1.2 (Fermentas, Landsmeer, The Netherlands). The resulting vector was digested with *Nco*I and *Xba*I and the excised fragment containing RLuc was exchanged with Firefly luciferase from the Kaiso reporter plasmid pGL3-4XKBS (Kelly et al., 2004a; a kind gift from Dr Juliet Daniel). Mouse mammary tumor cell lines were transfected with the reporter using Fugene HD (Promega) according to manufacturer’s instructions and assayed 48 h later for RLuc activity as described previously (Vermeulen et al., 2012). For normalization purposes, mCherry was co-transfected and expression was quantified using a fluorescence-activated cell sorting (FACS) analysis.

### RNA extraction, amplification and microarray hybridization

Total RNA was isolated from eight independent mILC cell lines grown either in the absence or presence of anchorage. Next, RNA was purified, reverse transcribed and amplified as described previously (Liu et al., 2007). Oligonucleotide microarrays containing 31,769 longmer probes representing 18,173 genes and 32,829 transcripts (Operon Biotechnologies Inc., Huntsville, AL) were a kind gift from the Netherlands Cancer Institute microarray facility. A total of 1 μg of amplified RNA was labeled with cyanine-5-conjugated ULS or cyanine-3-conjugated ULS (Kreatech Biotechnology, Amsterdam, The Netherlands). Hybridizations were processed, scanned and analyzed as described previously (Liu et al., 2007).

### GO term analysis

GO term analysis was performed using the Database for Annotation, Visualization and Integrated Discovery (DAVID) version 6.7 (http://david.abcc.ncifcrf.gov) as previously described (Huang et al., 2009).

### Motif matching and enrichment analysis

In order to determine whether differentially expressed genes were enriched for KBSs in their promoter regions, we performed a sequence matching procedure followed by an enrichment analysis. First, we obtained sequences for 1 kb upstream regions of all genes represented on the microarrays used in this study. In order to enrich for functional regions, we masked subsequences based on below-median sequence conservation levels across 18 Euarchontoglires. Conservation data were obtained from the UCSC Table Browser (Karolchik et al., 2004). Sequences were then scanned for matches with the Kaiso motif as present in the TRANSFAC database using a custom built C-program (Matys et al., 2006). A position was called a hit if the similarity between the sequence at that position and the Kaiso motif corresponded to at least 90% of the information content present in the motif. Upstream regions with at least one hit were regarded as putative Kaiso-binding targets. The total number of genes assayed was 24,787. Out of these, 1342 contained at least one KBS motif within their 1 kb promoter region. We defined a set of 249 differentially expressed genes based on a signal-to-noise ratio cutoff of 1.5 (supplementary material Table S1). From this we extracted 29 genes that presented a consensus KBS (Fig. 4C). Using the R statistical software package, we performed a Fisher’s Exact test to determine statistical significance (*P*<0.05) and corrected for multiple hypothesis testing.

### Chromatin immunoprecipitation

For chromatin immunoprecipitation (ChIP) experiments, 25×10^6^ cells were cross-linked, by adding formaldehyde directly to the medium to a final concentration of 1%, and were incubated at 37°C for 10 minutes. Cross-linking was inhibited with glycine (final concentration of 125 mM). Cells were put on ice and washed two times with ice-cold PBS. Cells were collected in 1 ml ice-cold lysis buffer [50 mM HEPES pH 7.8, 150 mM NaCl, 1 mM EDTA pH 8.0, 1% Triton, 0.1% SDS, 0.1% sodium deoxycholate and protease inhibitor tablets (cOmplete EDTA-free, Roche), pH 7.8], transferred to 1.5 ml Eppendorf tubes and subsequently centrifuged for 5 minutes at 2600 ***g*** at 4°C. Pellets were washed twice in 1 ml lysis buffer and sonicated in a Bioruptor (Diagenode, Denville, NJ) in ice water for 30 seconds per cycle (high settings, 30-second interval). Sonication efficiency (500–1000 bp) was verified using agarose gel electrophoresis. Samples were centrifuged for 5 minutes at 15,000 ***g*** after which the supernatant was transferred to a new Eppendorf tube. Anti-Kaiso antibody clone 6F or control IgG (4 μg each) was precoupled overnight to Protein A/G agarose beads (Thermo Scientific) in the presence of 200 μg sonicated herring sperm and 1.5% fish skin gelatin. Precoupled beads were incubated overnight with the sonicated chromatin in 500 μl lysis buffer. After centrifugation, the supernatant was removed and the beads were washed twice with lysis buffer, Wash Buffer 1 (250 mM NaCl in lysis buffer) and Wash buffer 3 (50 mM HEPES pH 7.8, 1 mM EDTA, 0.7% sodium deoxycholate, 1% NP-40, 0.5 M LiCl). Elution was performed by adding 130 μl of elution buffer (10 mM Tris-HCl pH 8.0, 1 mM EDTA, 1% SDS) to the beads and incubating the samples at 65°C overnight while shaking. Proteinase K was added to the samples and incubated at 37°C for 2 hours. Samples were centrifuged and the supernatant was purified using a column (Qiagen PCR purification kit). PCR was performed using primers flanking the most proximal KBS in the murine *Wnt11* promoter as described previously (Kim et al., 2004). For β-globin, the following primers were used: forward, 5′-CCCAGCGGTACTTTGATAGC-3′; reverse, 5′-GCCTTCACTTTGGCATTACC-3′.

### RNA isolation, cDNA synthesis and quantitative PCR

Cells were either cultured under adherent or non-adherent conditions and RNA was harvested after 24 hours using TRIzol^®^ reagent (Invitrogen, Bleiswijk, The Netherlands). cDNA was synthesized using iScript™ cDNA Synthesis Kit (BioRad, Veenendaal, The Netherlands) with 1 μg input RNA. Quantitative PCR was performed using the LightCycler^®^ 480 (Roche, Almere, The Netherlands) using the following primer sets: mouse Wnt11 (forward, 5′-CCAAGCCAATAAACTGATGCG-3′; reverse, 5′-GCATTTACACTTCGTTTCCAGGG-3′), mouse Kaiso (forward, 5′-CCAGCCTCTGTTGCTATTTCG-3′; reverse, 5′-GATTCACAGGAGTGGGAAGTTGA-3′), mouse GAPDH (forward, 5′-AAGCCCATCACCATCTTCC-3′; reverse, 5′-TAGACTCCACGACATACTCA-3′). For validation of *Wnt11*-knockdown PCR was performed using the primer sets as described above.

### Anoikis assay and colony formation assay

Anoikis resistance was determined and quantified as described previously (Derksen et al., 2006). In short, mILC cells were cultured in the presence of anchorage for 4 days and subsequently stained for propidium iodide (PI) and AnnexinV-FITC to assay for viability using FACS. Colony formation assays were performed as described previously (Schackmann et al., 2011).

### RhoA-GTP pulldown assays, cell fractionation, co-immunoprecipitation and western blotting

RhoA-GTP pulldown assays were performed as described previously (Schackmann et al., 2011). Cytosolic and nuclear fractions were derived using the REAP method and performed as described previously (Suzuki et al., 2010). mILC-1 cells were cultured in the presence of absence of anchorage for 24 hours before fractionation was performed. All samples were analyzed using SDS-PAGE and western blotting as described previously (Derksen et al., 2004). Co-immunoprecipitation of p120 was performed using mouse anti-p120 (4 μg for immunoprecipitation; BD Biosciences; 610134) coupled to Protein A/G agarose beads (Thermo Scientific). In short, cells were lysed for 20 minutes (20 mM Tris-HCl pH 7.5, 150 mM NaCl, 1 mM EDTA, 1 mM EGTA, 1% Trition X-100, 1 mM Na_3_VO_4_, 10 mM NaF, 5% glycerol). Lysates were pre-cleared with Protein A/G beads and incubated with the pre-coupled beads for 2 hours. Afterwards, beads were collected and washed three times with lysis buffer and subsequently eluted with sample buffer and boiled. The following antibodies were used: mouse anti-p120 clone 15D2 (1:2000; kind gift from Dr Albert Reynolds, Vanderbilt University, Nashville, TN), rabbit anti-Kaiso polyclonal (1:1000; kind gift from Dr Juliet Daniel, McMaster University, Hamilton, Canada), mouse anti-GAPDH (1:10,000; Millipore; mab374), rabbit anti-acetyl histon H3 (1:1000; Millipore; 06-599), goat anti-Akt1 (1:1000; Santa Cruz Biotechnology; sc-1618) and rabbit anti-RhoA (1:250; Santa Cruz Biotechnology; sc-179) antibodies.

## Supplementary Material

Supplementary Material
