# Supplementary material for: Nuclear p120-catenin regulates the anoikis resistance of mouse lobular breast cancer cells through Kaiso-dependent Wnt11 expression
Source: Dis Model Mech. 2015 Feb 20;8(4):373–84. doi: 10.1242/dmm.018648 (PMC4381336; doi:10.1242/dmm.018648)
Supplement: Supplementary Material [file supp_8_4_373__index.html]

Supplementary Material 

# Nuclear p120-catenin regulates the anoikis resistance of mouse lobular breast cancer cells through Kaiso-dependent Wnt11 expression

## DMM018648 Supplementary Material

**Files in this Data Supplement:**

- **Supplementary Material**
