# Supplementary material for: Nuclear p120-catenin regulates the anoikis resistance of mouse lobular breast cancer cells through Kaiso-dependent Wnt11 expression
Source: Dis Model Mech. 2015 Feb 20;8(4):373–84. doi: 10.1242/dmm.018648 (PMC4381336; doi:10.1242/dmm.018648)
Supplement: Supplementary Material [file supp_8.4.373_DMM018648.pdf]

Supplemental Table S1: Genes of the mILC Anoikis Resistance Transcriptome

0610031J06Rik, 1600029D21Rik, 1700055N04Rik, 1810011O10Rik, Abcc5, Abcg1, Abhd4, Acot1, Adssl1, Agt, Aldh1a3, Aldh2, Aldh3a1, Aldh3a2, Amigo1, Ank, Ankrd22, Aox1, App, Appl2, Arg1, Arg2, Arhgef3, Asprv1, Atp13a4, Atp6ap2, Atp6v0d1, BC016579, Bmf, C1qtnf1, C3, Calm4, Casp12, Casp4, Cbr2, Cd200, Cdh13, Ceacam2, Cfh, Cldn23, Clic3, Clu, Col6a1, Col6a2, Cp, Cpe, Crct1, Csad, Cst3, Cst6, Ctns, Ctsb, Ctsd, Ctsh, Cx3cl1, Cxcl5, Cyp1b1, Cyp4f13, Cyp4f39, Dcxr, Dhrs3, Dio2, Dmkn, Dnajb9, Dnase1l2, Dsc2, Ecm1, Efhd1, Ehf, Emb, Enpp2, Ephb6, Ephx1, Etnppl, Eva1a, Fabp4, Fam102a, Fam174a, Fam214a, Fetub, Foxq1, Fth1, Ftsj2, Fxyd2, Fxyd3, Gabrp, Gal3st1, Gbp2, Ghr, Gnptg, Gpa33, Gpr137b, Grhl1, Grina, Grn, Gsta1, Gsta3, Gsta4, Gstt3, H2-T23, Hbp1, Hdac11, Hist1h1c, Hist1h2bb, Hist1h2bc, Hist2h2aa1, Hmox1, Hs6st1, Hsd3b1, Id2, Ifi202b, Ifi203, Ifi205, Ifitm2, Igfbp3, Igfbp7, Il18, Il33, Inpp5k, Itm2b, Itpr2, Ivl, Klc4, Klk6, Klk7, Klk8, Krt14, Krt16, Krt6a, Krt71, Lamp1, Lamp2, Lce1a1, Lgr4, Lipa, Lrrc26, Ltbp3, Ltf, Lurap1l, Ly6a, Ly6d, Ly6g6c, Manba, Map1lc3b, Matn2, Mgst1, Mmp13, Mmp3, Mpp1, Mrpl4, Mt1, Mtss1, Mxd1, Mxra8, Mylip, Nipal2, Nrn1, Nt5e, Nudt7, Nupr1, Olfr701, Orm1, Orm2, P2rx4, Parm1, Pdcd4, Perp, Phyhd1, Pink1, Plcg2, Pld3, Pmp22, Prl2c3, Prl8a9, Prnp, Psca, Psd3, Ptprs, Rab11a, Rbp2, Rhbg, Rorc, S100a8, S100a9, Saa3, Sat1, Sbsn, Scara5, Scd1, Sdc2, Sdr42e1, Sectm1b, Serhl, Serinc3, Serpinb11, Sgcb, Slc1a4, Slc26a2, Slc43a2, Slc46a3, Slc5a1, Slurp1, Spaca4, Spp1, Sprr1a, Sprr1b, Sprr2a1, Sprr2d, Sprr2e, Sprr2i, Sprr3, Sqrdl, Sqstm1, St6gal1, Stap2, Sulf2, Sytl1, Tacstd2, Tbcel, Tc2n, Tgfb3, Tgm5, Thbs1, Timp2, Tlr2, Tmem176a, Tmem176b, Tmem86a, Tmprss11bnl, Tmprss11d, Tmprss2, Tmprss4, Tmprss6, Tpp1, Trex2, Trp63, Tslp, Ttc36, Ttc39c, Unc93b1, Vamp8, Wisp2, Wls, Wnt11, Xdh, Zbp1, Zdhhc14, Zfand2a, Zfand5

Supplemental Table S2: Enriched GO terms in mILC ART (ranked)

GO TERM	p-value
Keratinocyte differentiation	2,80E-07
Epidermal cell differentiation	4,60E-07
Epithelial cell differentiation	1,10E-06
Epidermis development	1,30E-06
Ectoderm development	2,40E-06
Regulation of programmed cell death	3,00E-04
Regulation of cell death	3,20E-04
Epithelium development	4,10E-04
Induction of apoptosis	5,50E-04
Induction of programmed cell death	5,50E-04
Regulation of apoptosis	7,10E-04
Positive regulation of apoptosis	7,10E-04
Positive regulation of programmed cell death	7,60E-04
Positive regulation of cell death	8,10E-04
Oxidation reduction	1,10E-03
Response to wounding	3,40E-03
Inflammatory response	4,30E-03

Ion homeostasis	8,00E-03
Proteolysis	9,10E-03
Positive regulation of developmental process	1,00E-02
Cellular ion homeostasis	1,10E-02
Chemical homeostasis	1,30E-02
Cellular chemical homeostasis	1,30E-02
Homeostatic process	1,60E-02
Negative regulation of programmed cell death	2,10E-02
Cell adhesion	2,50E-02
Biological adhesion	2,50E-02
Regulation of cell proliferation	3,80E-02
Cellular homeostasis	5,00E-02
Immune response	6,50E-02

Supplemental Table S3: Transcription factor enrichment in mILC ART ranked by significance

TRANSFAC matrix ID	p-value	Hits in mILC ART	Total hits
V$HMGIY_Q6-Unknown	9.43889E-09	187	9763
V$P53_02-p53	3.45435E-09	99	4227
V$MYOD_Q6-MyoD	3.45435E-09	104	4516
V$SREBP1_Q6-SREBP-1	1.14755E-09	99	4122
P$PBF_Q2-PBF	9.40225E-08	182	9121
V$CACBINDINGPROTEIN_Q6-Unknown	7.50152E-08	147	6884
V$AREB6_04-AREB6	5.01061E-08	176	8645
V$AR_Q6-AR	2.46904E-08	177	8621
V$MYOGENIN_Q6-myogenin	1.12845E-08	87	3256
V$MAZ_Q6-MAZ	4.91751E-07	112	4544
V$E2A_Q6-E2A	4.25357E-07	101	3917
F$STRE_B-STRE	2.10015E-07	154	6894
V$STAT3_02-STAT3	3.10307E-06	256	13428
V$STAT1_03-STAT1	2.59977E-06	239	12162
V$GR_Q6_01-GR	2.59977E-06	210	10205
V$HNF4_Q6_02-HNF4	7.72736E-05	242	12201
V$HNF4_Q6_03-HNF4	2.19221E-05	272	14187
I$DL_02-dl	0.006123211	60	2611
V$ZTA_Q2-Zta	0.006253739	37	1383
V$STAT1_02-STAT1	0.006263223	143	7631
V$TAL1_Q6-TAL1	0.006282561	51	2126
V$CEBP_Q3-CEBP	0.006979195	100	4976

I$GAGAFACTOR_Q6-Unknown	0.007244069	93	4578
NRSE_REST_PART2	0.007244069	89	4336
V$GATA4_Q3-GATA-4	0.007244069	35	1306
V$NFE2_01-NF-E2	0.007244069	20	587
V$P53_DECAMER_Q2-Unknown	0.007244069	88	4273
V$ZNF219_01-ZNF219	0.007244069	61	2701
P$AGP1_01-AGP1	0.008103067	35	1318
V$FREAC2_01-bending	0.009168778	7	99
V$NFKB_Q6_01-NF-kappaB	0.011314379	16	437
I$TTK69_01-Unknown	0.011405196	50	2152
I$SRYBETA_Q6-Sry-beta	0.011617357	18	527
V$TFIII_Q6-TFII-I	0.011617357	107	5517
V$NFY_Q6_01-NF-Y	0.012274504	45	1891
NRSE_REST_PART1	0.012613969	26	908
V$COREBINDINGFACTOR_Q6-Unknown	0.013662913	63	2915
I$BCD_01-Bcd	0.014689811	16	454
V$USF_C-USF	0.015966955	68	3228
N$SKN1_01-Skn-1	0.016684756	26	931
N$UNC86_Q2-unc-86	0.019252744	20	653
V$LFA1_Q6-LF-A1	0.019252744	63	2966
V$MYB_Q6-c-Myb	0.019252744	75	3673
V$FOX_Q2-Unknown	0.019346618	31	1200
V$MTF1_Q4-MTF-1	0.020384769	7	119
V$OCT1_06-Oct-1	0.020475716	31	1207
V$SMAD3_Q6-SMAD-3	0.020690145	42	1797

F$STE12_Q4-STE12	0.025580219	60	2840
V$KAISO_01-KAISO	0.028043811	29	1342
V$T3R_01-v-ErbA	0.029045111	6	96
V$PADS_C-Unknown	0.033181267	39	1685
V$HELIOSA_02-Unknown	0.034052561	56	2653
V$PEA3_Q6-PEA3	0.034052561	57	2712
V$STAT_Q6-STAT	0.034052561	55	2597
V$HEB_Q6-HEB	0.034238326	43	1916
V$BARBIE_01-Unknown	0.034289088	6	101
V$IRF_Q6_01-IRF	0.036730654	62	3021
V$PEBP_Q6-PEBP	0.040749782	8	176
F$RCS1_Q2-RCS1	0.042242494	5	75
P$SED_Q2-SED	0.042242494	48	2232
V$AML1_Q6-AML1	0.042242494	33	1394
V$E2F1_Q3-E2F-1	0.042824793	154	8834
I$SN_01-Sn	0.046056884	21	777
V$AREB6_02-AREB6	0.046056884	30	1248
V$CDC5_01-Cdc5	0.046056884	7	146
V$E4BP4_01-E4BP4	0.046056884	8	182
V$IRF1_Q6-IRF1	0.046056884	66	3310
V$NFKAPPAB65_01-Unknown	0.046056884	22	827
I$BRCZ4_01-Unknown	0.049446006	51	2437
V$E47_01-E47	0.049446006	16	539
